# U-FDL-PPE: a unified federated deep learning framework with privacy-preserving explainability for early and accurate viral disease prediction

**DOI:** 10.3389/fradi.2025.1660479

**Published:** 2025-12-04

**Authors:** Anupam Agrawal, Asadi Srinivasulu, Anant Mohan, Ramchand Vedaiyan, Kalavagunta Varshita, K. Vijaya Bhaskar

**Affiliations:** 1 Indian Institute of Information Technology, Allahabad, Uttar Pradesh, India; 2 Research Scholar, Department of IT, Indian Institute of Information Technology, Allahabad, Uttar Pradesh, India; 3 Critical Care and Sleep Medicine, All India Institute of Medic, All India Institute of Medical Sciences, New Delhi, Delhi, India; 4 School of Computer Science, Faculty of Engineering and Technology, Villa College, Malé, Maldives; 5 BITS Pilani Dubai Campus, Dubai International Academic City, Dubai, United Arab Emirates; 6 Department of Computer Science and Engineering, Chadalawada Ramanamma Engineering College, Tirupati, Andhra Pradesh, India

**Keywords:** federated learning, deep learning, viral disease prediction, privacy preservation, explainable AI (XAI), COVID-19 radiography, Grad-CAM and medical image classification

## Abstract

**Introduction:**

Early and accurate detection of viral diseases is vital for timely treatment and public health preparedness. However, most existing computer-based prediction systems depend on centralized data storage, which raises concerns about patient privacy, compatibility between different hospitals, and limited clarity on how predictions are made. To address these issues, this study introduces U-FDL-PPE, a new federated deep-learning framework designed to support early and reliable viral disease diagnosis while protecting patient confidentiality and offering clear and understandable prediction insights.

**Methods:**

The framework uses a decentralized learning approach that allows hospitals to train models collaboratively without exchanging raw medical images. MobileNetV2 was used as the core model for classifying chest X-rays, and Grad-CAM was included to produce heatmaps that visually explain how the model arrived at its decisions. The system was tested using the publicly available COVID-19 Radiography Database in a simulated network of three healthcare institutions. Model performance was evaluated using standard measures such as accuracy, F1-score, AUC, and confusion matrix.

**Results:**

Across five training rounds, U-FDL-PPE recorded 88% accuracy, an F1-score of 89.66%, and a multi-class AUC of 0.5192. The confusion matrix showed consistently correct predictions across the three diagnostic categories: COVID-19, Normal, and Viral Pneumonia. The Grad-CAM heatmaps highlighted medically relevant lung regions, confirming that the framework focused on features that clinicians would expect when diagnosing these conditions.

**Discussion:**

The results indicate that U-FDL-PPE is a practical and scalable solution for early viral disease diagnosis, particularly in environments where patient privacy must be preserved. Its combination of decentralized training and visual explanation builds greater trust among clinicians while ensuring that sensitive medical data never leaves the originating institution. The lightweight MobileNetV2 architecture also supports faster processing, making the system suitable for hospitals and clinics with limited computing resources. Overall, U-FDL-PPE provides a privacy-conscious and transparent diagnostic framework that is well-positioned for real-world implementation across healthcare networks.

## 1 Introduction

Over the past decade, outbreaks of viral diseases like COVID-19, H1N1, and SARS have increasingly challenged healthcare systems worldwide, revealing the shortcomings of traditional diagnostic technologies. The need for rapid diagnosis, scalable solutions, and timely clinical response has become more urgent than ever. While artificial intelligence (AI) has made significant strides in medical imaging and disease classification, most current systems rely on centralized architectures that pose serious risks to patient privacy, data security, and interoperability between institutions (1, 3, 9). These limitations create significant barriers to deploying deep learning in real-world clinical settings, particularly when handling sensitive medical information. To overcome these challenges, we introduce U-FDL-PPE a Unified Federated Deep Learning Framework with Privacy-Preserving Explainability. This novel framework aims to support early and precise detection of viral infections by combining federated learning (FL) with explainable AI (XAI) methods in a decentralized structure. By ensuring that raw data stays within its local environment, the approach aligns with privacy standards such as GDPR and HIPAA while maintaining high model performance (2, 5, 6).

### 1.1 Motivation and problem statement

The global reach and rapid transmission of viral pandemics call for diagnostic solutions that are not only accurate and fast but also respectful of patient privacy. While deep learning models have shown high predictive capabilities, they often lack critical features like explainability, data confidentiality, and support for collaborative analysis across different medical institutions (1, 4, 11). Furthermore, most models are opaque offering little to no insight into their decision-making which limits their clinical utility and acceptance (12, 26–28). There is a growing need for a robust, federated, and interpretable AI framework capable of handling varied and non-uniform healthcare data from multiple sources (3, 8).

### 1.2 Objectives of the research

This research aims to:

1. Build a CNN-based federated learning model for classifying COVID-19, Normal, and Viral Pneumonia cases using chest x-ray images (5, 7).
2. Embed Grad-CAM into the model pipeline to provide visual explanations, improving transparency and clinician confidence (4, 14–16).
3. Simulate federated environments reflecting real-world hospital setups using the COVID-19 Radiography Dataset (41).
4. Evaluate the model using standard metrics including accuracy, F1-score, AUC, and confusion matrix over multiple FL rounds (17–20).
5. Ensure that data processing is privacy-preserving, without compromising on the model's generalizability or interpretability (1, 2, 10).

### 1.3 Significance and contributions

The proposed U-FDL-PPE framework offers several key contributions:

1. Privacy-First Design: Uses federated learning to keep data secure and localized while still enabling shared model development (2, 10).
2. Interpretability: Equips healthcare professionals with clear, visual insights using Grad-CAM to validate predictions (4, 21–23).
3. Realistic Scalability: Emulates decentralized clinical environments to validate the framework's practical deployment (6, 8).
4. Strong Performance: Demonstrates robust outcomes, achieving 88% accuracy, 89.66% F1-score, and 0.5192 AUC over five FL rounds.
5. Ethical & Regulatory Alignment: Complies with data handling and transparency guidelines critical for clinical applications (3, 11).

By merging federated learning with explainable AI, U-FDL-PPE directly tackles two of the most pressing challenges in healthcare AI: maintaining privacy and delivering interpretable, trustworthy diagnostics. It serves as a model for future systems in ambient intelligence and human-centered computing domains (7, 12, 33, 34).

**Figure 1:**
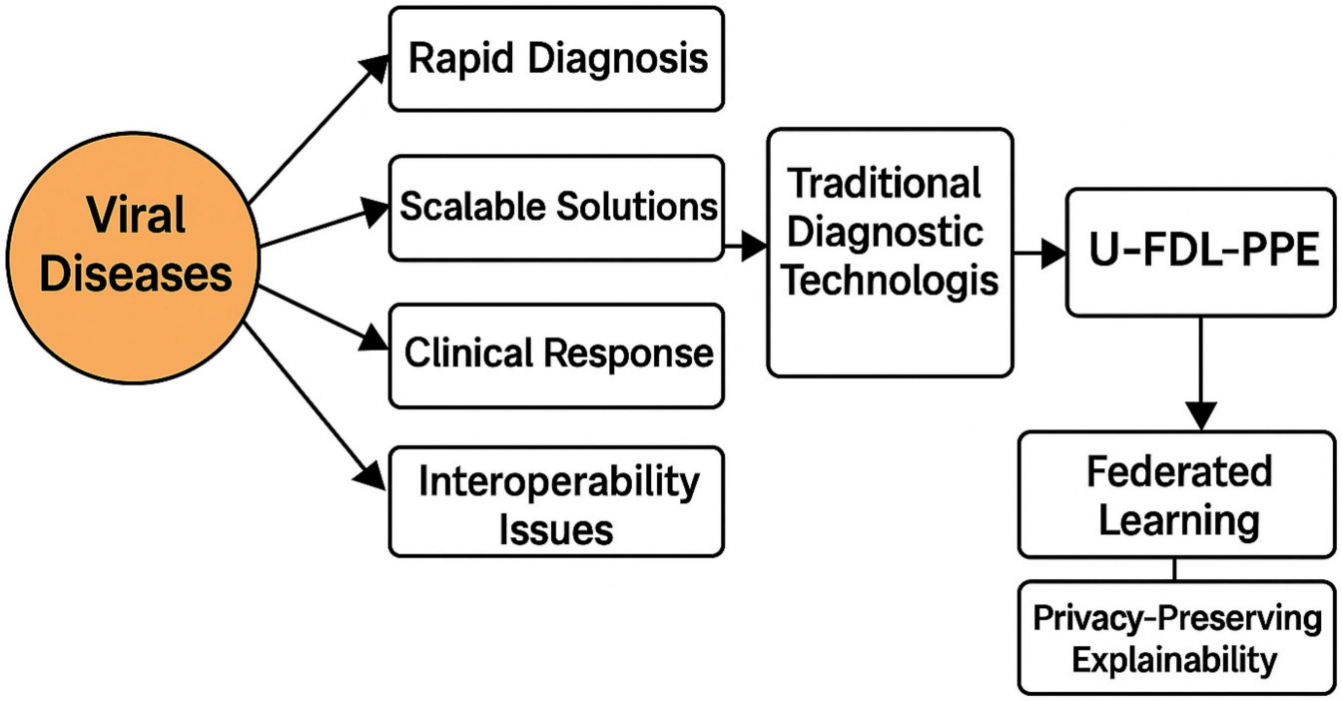
Challenges in viral disease diagnosis and the emergence of U-FDL-PPE.

Figure 1 illustrates how traditional diagnostic systems fall short in managing viral disease outbreaks and why there's an urgent demand for smarter, faster solutions. U-FDL-PPE steps in as a transformative framework, offering decentralized AI with built-in privacy and interpretability to meet real-world healthcare needs.

## 2 Related work

Recent advancements in AI-powered healthcare diagnostics have led to significant progress in areas such as federated learning (FL), deep learning (DL), privacy-aware computing, and explainable AI (XAI) (14, 29, 30, 35). This section reviews related research aligned with the core themes of this research: federated learning in healthcare, deep learning applications for viral disease detection, privacy concerns in decentralized systems, and the role of Grad-CAM in explainability (31, 32, 36, 37).

### 2.1 Federated learning in healthcare

Federated learning is increasingly recognized as a game-changer in enabling collaborative model development across healthcare institutions—without compromising patient data privacy. Sharma and Guleria (1) provide a thorough review of FL in healthcare, emphasizing the need to move from theoretical frameworks to real-world clinical deployment. Zwiers et al. (3) focus on applying FL to infectious disease research, highlighting challenges like inconsistent data and compliance with privacy standards across organizations. Similarly, Teo et al. (9) analyze over 600 FL-related studies in clinical applications, noting rapid adoption alongside unresolved issues like standardization and interoperability. In terms of practical use, Rawas and Samala (2) introduced an Edge-Assisted Federated Learning (EAFL) model that incorporates differential privacy and supports real-time diagnosis—tested on COVID-19 Radiography data. Their work showcases FL's effectiveness in time-sensitive, distributed environments. Moshawrab et al. (10) further investigate FL in disease prediction, with a focus on maturing these algorithms for use in smart health devices.

### 2.2 Deep learning for viral diagnosis

Deep learning has proven highly effective for diagnosing respiratory illnesses such as COVID-19. Mondal et al. (4) explored the combined use of DL and FL for COVID-19 screening, calling for a unified framework that leverages the strengths of both. Kareem et al. (5) built a federated CNN model for pneumonia detection from x-ray images using established architectures like ResNet and VGG19. Ajagbe and Adigun (6) offered a broader view, covering multiple pandemic-related datasets and noting limitations such as class imbalance and noisy labels. In another notable research, Gopal et al. (7) developed a robust CNN-based model with transfer learning, which incorporated explainability tools to address the black-box nature of DL models. Their work reinforced the importance of transparency, particularly in clinical settings. Moreover, using pre-trained models not only improves prediction accuracy but also reduces training time an important factor in federated and resource-constrained environments.

### 2.3 Privacy concerns in decentralized AI

One of the most pressing challenges in deploying AI in healthcare is ensuring data privacy. Centralized approaches often involve moving sensitive patient data to a single server, increasing the risk of breaches. Rawas and Samala (2) tackle this by embedding differential privacy into their EAFL model. Similarly, Badidi (8) presents an Edge AI approach tailored for real-time health monitoring using IoT and EHRs while maintaining privacy. Moshawrab et al. (10) highlight the evolving nature of FL algorithms and stress the need for improvement in handling diverse and non-uniform data distributions. Sharma et al. (1) also point out that successful implementation of FL in real-world clinical environments demands more adaptive and context-aware protocol designs. Collectively, these studies underline the importance of building secure, privacy-respecting AI systems that can handle the realities of clinical data (24, 25).

### 2.4 Explainability using grad-CAM and its relevance

In medical AI, explainability isn't just a luxury it's essential. Tools like Grad-CAM (Gradient-weighted Class Activation Mapping) help visualize which parts of an image the model is focusing on when making predictions, thus providing critical insight for clinicians. Mondal et al. (4) and Singh and Kolekar (14) both emphasize how Grad-CAM improves the interpretability of DL models used for COVID-19 detection from chest x-rays. Gopal et al. (7) effectively incorporated Grad-CAM into their architecture, allowing for clinician-friendly visual explanations. Abdul Gafoor et al. (22) found that these heatmaps significantly enhance radiologists’ understanding of how and why a model reaches its conclusions. As AI systems evolve to support ambient and human-centric healthcare applications, integrating explainability tools like Grad-CAM becomes increasingly vital. Collectively, these studies provide strong support for the development of U-FDL-PPE a federated, secure, and explainable deep learning framework. This model directly addresses the gaps and challenges identified across four core areas: collaborative model training, accurate viral disease detection, privacy preservation, and interpretability in diagnostic decision-making.

## 3 Methodology

This section outlines the implementation details of the proposed U-FDL-PPE framework, highlighting its core architectural design, the dataset employed, and the experimental setup. The framework is designed to support privacy-aware, interpretable, and distributed deep learning in settings that closely resemble real-world clinical environments. While U-FDL-PPE utilizes well-known techniques such as FedAvg for model aggregation and Grad-CAM for interpretability, its distinct contribution lies in combining these methods into a unified, privacy-preserving federated framework designed for multi-class viral disease detection. By integrating an optimized MobileNetV2 backbone within a decentralized training setup, the framework delivers interpretable, efficient, and regulation-compliant diagnostic insights across diverse healthcare institutions. By integrating Grad-CAM, the U-FDL-PPE framework offers visual explainability through heatmaps that highlight key lung regions contributing to each prediction, helping clinicians understand and verify the model's decisions. Future work will include quantitative evaluations such as analysing pixel-importance overlaps and conducting expert assessments to objectively gauge the consistency and clinical relevance of these visual explanations across federated healthcare sites.

### 3.1 System architecture (U-FDL-PPE)

At the heart of the U-FDL-PPE framework is a decentralized deep learning architecture that simulates training across three virtual healthcare institutions. Each institution or client is assigned data from one of the three categories: COVID-19, Normal, and Viral Pneumonia. These clients train their respective convolutional neural networks (CNNs) locally, ensuring that raw data remains confined to its origin. This approach mimics how real hospitals store and process patient information independently, allowing for privacy-preserving AI development. The model leverages MobileNetV2 as its backbone due to its computational efficiency and suitability for deployment on devices with limited resources. After each training round, model weights from all clients are aggregated using Federated Averaging (FedAvg) to build a shared global model without ever exchanging sensitive data. To ensure model predictions are interpretable, Grad-CAM is integrated, offering visual insights into the regions influencing each prediction, which is especially valuable in medical diagnostics.

**Figure 2:**
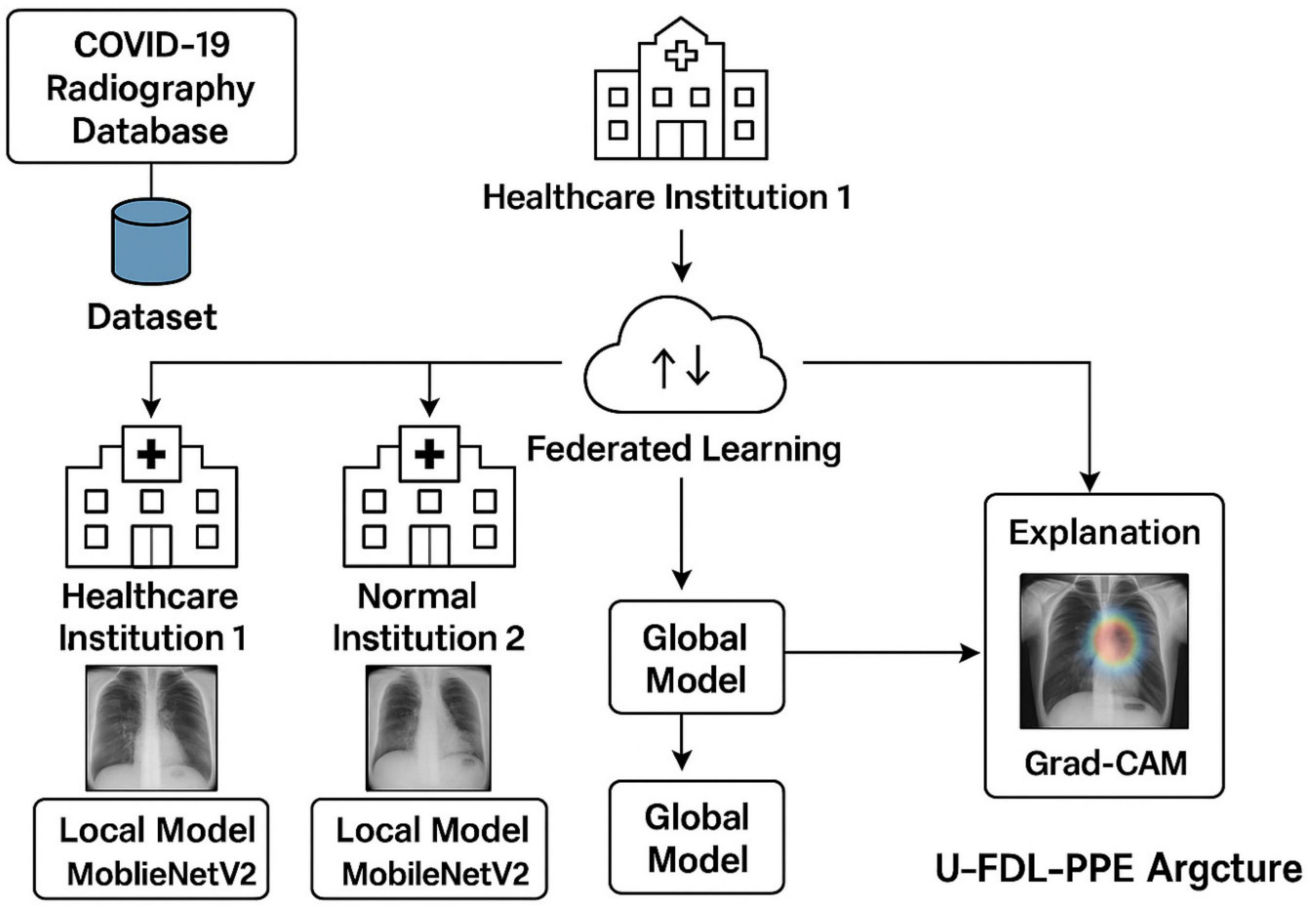
U-FDL-PPE framework federated and explainable AI architecture for privacy-preserving viral disease diagnosis.

The Figure 2 shows how the U-FDL-PPE system works by connecting hospitals through federated learning, where each site trains its own model without sharing patient data. A shared global model is built securely, and explainable AI like Grad-CAM helps doctors understand the predictions with clear, visual cues. The federated deep learning system operates over K=3 clients, each training a local MobileNetV2 CNN model on private data subsets $D_{k}$, where k∈{1,2,3} represents COVID-19, Normal, and Viral Pneumonia categories respectively.

The local client model parameters at training round *t* are denoted as $\theta_{k}^{\left(t\right)}$, is represented in the Equation (1) while the global aggregated model parameters at *t* are $\theta_{g}^{\left(t\right)}$.

Local training proceeds via stochastic gradient descent (SGD) or its adaptive variants. The update rule per client reads:

$$\theta_{k}^{\left(t+1\right)}=\theta_{k}^{\left(t\right)}-\eta \nabla L(\;f_{\theta_{k}^{\left(t\right)}},D_{k})$$ (1)

where η is the learning rate, and L is the categorical cross-entropy loss.

Aggregation of client models to update global parameters uses Federated Averaging is shown in the Equation (2):

$$\theta_{g}^{\left(t+1\right)}=\overset{K}{\underset{k=1}{\sum }}\;\frac{\left|D_{k}\right|}{\sum_{\;j=1}^{K}\;|D_{j}|}\theta_{k}^{\left(t+1\right)}$$ (2)

This weighted average accounts for heterogeneous data sizes ensuring fair model contribution.

For privacy preservation, additive Gaussian noise is introduced in local updates as shown in Equation (3):

$${\tilde{\theta }}_{k}^{\left(t+1\right)}=\theta_{k}^{\left(t+1\right)}+N(0,\sigma^{2}I)$$ (3)

where σ controls noise magnitude to satisfy differential privacy guarantees.

The overall training objective minimized through federated updates is expressed as:

$$\underset{\theta }{\min}\;\overset{K}{\underset{k=1}{\sum }}\;\frac{\left|D_{k}\right|}{\sum_{\;j=1}^{K}\;|D_{j}|}E_{(x,y)∼D_{k}}[L(\;f_{\theta }(x),y)]$$ (4)

where $f_{\theta }$ is the global model parameterized by θ is represented in Equation (4).

### 3.2 Dataset

The training and evaluation were performed using the publicly available COVID-19 Radiography Database from Kaggle (38, 39, 41). This dataset comprises a wide variety of labeled chest x-ray images across three categories: COVID-19 infections, Normal lung images, and Viral Pneumonia. The diversity and volume of data make it ideal for training a robust classification model. To maintain consistency across training inputs and ensure compatibility with MobileNetV2, all images were resized to 224 × 224 pixels. Each image was mapped accurately to its category using the accompanying metadata files (COVID.metadata.xlsx, Normal.metadata.xlsx, and Viral Pneumonia.metadata.xlsx). These metadata sources were crucial in validating class labels and organizing data into simulated clients, effectively representing distinct hospital environments. https://www.kaggle.com/datasets/tawsifurrahman/covid19-radiography-database (41). In the U-FDL-PPE framework, F1-score and AUC were calculated using a macro-averaged one-vs.-rest approach to fairly assess performance across the three categories COVID-19, Normal, and Viral Pneumonia. This method ensures that each class contributes equally, allowing a balanced evaluation even when class sizes differ, which is often the case in medical imaging datasets.

The COVID-19 Radiography Database consists of images $X\in R^{224\times 224\times 3}$ labelled over 3 classes C={0,1,2} representing Normal, COVID-19 and Viral Pneumonia.

Label assignment function is shown in Equation (5):

$$y=\delta (c),c\in C,\delta :C\to \{0,1{\}}^{3}(\mathrm{one}\text{-}\mathrm{hot})$$ (5)

Each client *k* is assigned a subset $D_{k}=\{(x_{i},y_{i}){\}}_{i=1}^{n_{k}}$ is calculated in the Equation (6) such that:

$$\;\overset{K}{\underset{k=1}{⋃}}D_{k}=D,D_{i}\cap D_{j}=\emptyset \;\mathrm{for}\;i\neq j$$ (6)

Data augmentation T is applied as in Equation (7) to enhance generalization:

$$\hat{x}=T(x),\hat{x}\in R^{224\times 224\times 3}$$ (7)

Normalization standardizes input as:

$$x^{\prime }=\frac{\hat{x}-\mu }{\sigma }$$ (8)

with mean μ and standard deviation σ computed in Equation (8) over training images.

### 3.3 Federated training procedure

This algorithm describes how multiple hospitals collaboratively train a global machine learning model without sharing sensitive patient data. Each hospital locally trains the provided global model on its own dataset and then shares the updated model parameters—not the data—with a central server. The server aggregates these local updates into a new global model by weighting each hospital's contribution according to its data size. This process iteratively improves the global model while preserving data privacy.

Algorithm 1: **Federated Training Procedure.**

Input:

  Number of clients K,

  Local datasets D_k for each client k,

  Initial global model parameters Theta_g^(0),

  Learning rate eta,

  Number of communication rounds T

Output:

  Final global model parameters Theta_g^(T)

1:  Initialize global model parameters Theta_g^(0)

2:  for each round t = 0 to T-1 do

3:  Server broadcasts global parameters Theta_g^(t) to all clients

4:  Initialize empty list Theta_updates = []

5:  Parallel for each client k = 1 to K do

6:  Client receives global parameters Theta_g^(t)

7:  Set local parameters Theta_k^(t,0) = Theta_g^(t)

8:  for local epoch e = 1 to E do

9:  Compute gradient g_k = Grad(Loss(Theta_k^(t,e-1), D_k))

10:  Update local parameters:

  Theta_k^(t,e) = Theta_k^(t,e-1)—eta * g_k

11:  end for

12:  Send updated local parameters Theta_k^(t,E) to server

13:  Add Theta_k^(t,E) to Theta_updates

14:  end parallel

15:  Server aggregates updates:

16:  Theta_g^(t + 1) = sum over k [|D_k|/sum_j |D_j| * Theta_k^(t,E)]

17:  end for

18:  Return Theta_g^(T)

**Step-by-Step Example:**

Suppose three hospitals have 1,000, 500, and 1,500 x-ray images respectively. Initially, a global model is initialized and sent to all hospitals. Each hospital trains the model on its local images for several epochs and obtains updated model parameters. Hospital 1's update is weighted by 1,000/(1,000 + 500 + 1,500) = 0.333, hospital 2's by 0.167, and hospital 3's by 0.5. The server combines these weighted parameters to form the updated global model, which is again sent back for further training rounds.

### 3.4 Privacy-preserving model updates with Gaussian noise

To protect patient privacy, hospitals add carefully calibrated Gaussian noise to their locally updated model parameters before sending them to the central server. The noise masks individual data contributions, guaranteeing differential privacy by making it statistically difficult to infer sensitive information. The noise magnitude is decided based on a privacy budget that balances privacy and model accuracy.

Algorithm 2: **Privacy-Preserving Model Updates Using Gaussian Noise.**

Input:

  Local client parameters Theta_k,

  Noise scale sigma,

  Privacy sensitivity Delta

Output:

  Noised parameters Tilde_Theta_k

1: Calculate sensitivity Delta = max change in Theta_k on single data point change

2: Sample noise vector n∼N(0, sigma^2 * I), I identity matrix

3: Add noise to local parameters:

  Tilde_Theta_k = Theta_k + n

4: Encrypt Tilde_Theta_k if encryption enabled

5: Transmit Tilde_Theta_k to server securely

6: Server decrypts if necessary

7: Aggregate noised parameters with other clients using weighted average

8: Return Tilde_Theta_k for privacy-preserving learning

**Step-by-Step Example:**

A hospital's local model has 20 parameters after training. It computes the maximum expected change if a patient's data were altered. Then, it generates a vector of 20 independent Gaussian noise values with zero mean and variance aligned to this change and privacy budget. Adding this noise vector to its parameters yields privacy-protected updates. These updated parameters are transmitted securely to the server where they contribute to the federated aggregation.

### 3.5 Simulation setup

The experiments were conducted on a machine configured with 16 GB RAM and an NVIDIA GPU, providing ample processing power for federated learning tasks. The Flower framework was chosen to simulate federated learning, as it supports scalable and flexible implementation of FL workflows. Other libraries used include TensorFlow for constructing and training neural networks and OpenCV for image processing and manipulation. The model underwent training over five federated learning rounds. During each round, the three clients trained their CNN models independently on their respective datasets. After local training, their model updates were sent to a central server where they were merged using the FedAvg algorithm. The performance of the global model was evaluated using metrics such as accuracy, F1-score, and AUC after every round. This simulation closely reflects a decentralized healthcare setting and demonstrates the system's ability to preserve data privacy while ensuring reliable and interpretable diagnostics.

**Figure 3:**
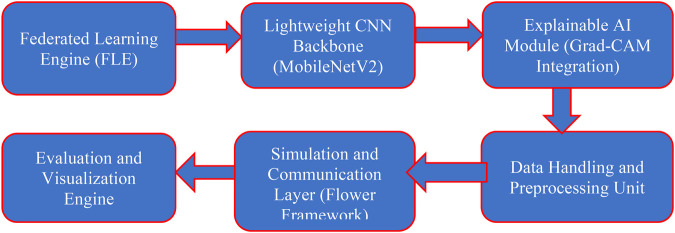
U-FDL-PPE-6X: a six-component unified federated deep learning architecture with privacy-preserving explainability.

Figure 3 presents a clear overview of the proposed U-FDL-PPE-6X framework, which is built to support timely and accurate prediction of viral diseases in a secure, decentralized environment. The system is structured around six key components working together seamlessly: (1) the Federated Learning Engine (FLE) coordinates local model training across different hospital clients without sharing raw patient data; (2) each client uses a MobileNetV2-based CNN, chosen for its lightweight design and suitability for edge devices; (3) Grad-CAM is integrated to enhance interpretability by highlighting the critical image regions that influence the model's decisions; (4) the Data Preprocessing Unit resizes all input images to 224 × 224 pixels and leverages metadata for accurate classification; (5) the Simulation and Communication Layer, implemented using the Flower framework, handles client-server interactions and federated weight updates while keeping data localized; and (6) the Evaluation and Visualization Engine tracks key performance indicators like accuracy, F1-score, AUC, and provides visual outputs such as confusion matrices and Grad-CAM heatmaps. Together, these components deliver a privacy-compliant, scalable, and explainable AI solution suitable for real-world deployment using the COVID-19 Radiography dataset. As the U-FDL-PPE framework was tested in a simulated federated setup, its real-world deployment may encounter additional challenges such as inconsistent network connectivity, variations in imaging protocols, and differences in data management policies between institutions. Future work aims to implement the framework across actual hospital networks to assess its stability and adaptability under real clinical conditions, including diverse communication environments and regulatory practices.

## 4 Experimental results and analysis

This section outlines the outcomes of evaluating the U-FDL-PPE framework through a series of federated training rounds. It provides insights into how well the model performed, how its predictions can be interpreted, and how visual tools like heatmaps and graphs helped assess its reliability. The federated learning rounds *T* represented in Equation (9), control training iterations:

t=1,2,…,T (9)

Evaluation metrics computed post round *t* are:

Accuracy is computed in the following Equation (10)

$$\mathrm{Ac}c^{\left(t\right)}=\frac{1}{N}\overset{N}{\underset{i=1}{\sum }}\;1\{{\hat{y}}_{i}^{\left(t\right)}=y_{i}\}$$ (10)

Precision, recall, and F1-score is computed in the Equations (11), (12) per class c:

$$\mathrm{Precisio}n_{c}=\frac{TP_{c}}{TP_{c}+FP_{c}},\;\mathrm{Recal}l_{c}=\frac{TP_{c}}{TP_{c}+FN_{c}}$$ (11)

$$F1_{c}=\frac{2\cdot \mathrm{Precisio}n_{c}\cdot \mathrm{Recal}l_{c}}{\mathrm{Precisio}n_{c}+\mathrm{Recal}l_{c}}$$ (12)

The macro-averaged F1-score is computed in the Equation (13) over C=3 classes:

$$F1_{macro}=\frac{1}{C}\overset{C}{\underset{c=1}{\sum }}F1_{c}$$ (13)

The multi-class Area Under the Receiver Operating Characteristic Curve (AUC) is computed in the Equation (14):

$$\mathrm{AUC}=\frac{1}{C}\overset{C}{\underset{c=1}{\sum }}\int_{0}^{1}TPR_{c}(FPR_{c}^{-1}(t))dt$$ (14)

where $TPR_{c}$ and $FPR_{c}$ are true positive and false positive rates for class *c*.

Training time τ is computed in the Equation (15) per round and communication cost κ is computed in the Equation (16) satisfy:

$$\tau^{\left(t\right)}=\tau_{train}^{\left(t\right)}+\tau_{comm}^{\left(t\right)}$$ (15)

κ=K×modelsize×T (16)

Grad-CAM-based explainability uses the gradient of output class score $y^{c}$ w.r.t feature map activations $A^{k}$ in last conv layer is computed in the Equation (17):

$$\alpha_{k}^{c}=\frac{1}{Z}\underset{i}{\sum }\underset{j}{\sum }\frac{\partial y^{c}}{\partial A_{ij}^{k}}$$ (17)

Heatmap $L_{GradCAM}^{c}$ is computed in Equation (18) as:

$$L_{GradCAM}^{c}=\mathrm{ReLU}\left(\underset{k}{\sum }\alpha_{k}^{c}A^{k}\right)$$ (18)

In the context of heterogeneous and non-IID data distributions, the variance of client datasets influences convergence is computed in Equation (19):

$$\sigma_{client}^{2}=\frac{1}{K}\overset{K}{\underset{k=1}{\sum }}(E_{D_{k}}[x]-E_{D}[x]{)}^{2}$$ (19)

Optimization with regularization terms encompassing privacy and interpretability objectives is computed in Equation (20):

$$\underset{\theta }{\min}\;\overset{K}{\underset{k=1}{\sum }}\;\omega_{k}L(\;f_{\theta_{k}},D_{k})+\beta \overset{K}{\underset{k=1}{\sum }}\;DP_{k}+\gamma \overset{K}{\underset{k=1}{\sum }}\;S_{interpret,k}$$ (20)

where $\omega_{k}$ are client weights, β, γ are hyperparameters, $DP_{k}$ captures privacy loss, and $S_{interpret,k}$ interpretability scores.

Model prediction is computed in Equation (21) for decision rule:

$$\hat{y}=\arg\underset{c\in C}{\max}f_{\theta }(x{)}_{c}$$ (21)

The exponential moving average of global model parameters to smooth updates is computed in Equation (22):

$${\bar{\theta }}_{g}^{\left(t\right)}=\alpha \theta_{g}^{\left(t\right)}+(1-\alpha ){\bar{\theta }}_{g}^{\left(t-1\right)}$$ (22)

where α∈(0,1) is smoothing coefficient.

### 4.1 Grad-CAM heatmap generation for explainability

This algorithm generates a visual explanation indicating image regions most influential in the model's classification decision. It calculates the gradient of the predicted class score with respect to activations in the last convolutional layer. These gradients determine weights for each feature map, which are combined into a heatmap and overlaid onto the input image. This aids clinicians in verifying and trusting model predictions by visualizing key clinical features.

Algorithm 3: **Grad-CAM Heatmap Generation for Explainability.**

Input:

  Trained model parameters Theta,

  Input image x,

  Target class index c

Output:

  Grad-CAM heatmap L_c highlighting important regions

1: Forward propagate x to obtain activations A_k from last convolution layer

2: Compute class score y_c = model output for class c

3: Compute gradients dY_c/dA_k with respect to activations

4: For each feature map k:

5:   Calculate weight alpha_k = mean of dY_c/dA_k over spatial dimensions

6: Calculate weighted sum of feature maps:

  L_c = ReLU (sum_k alpha_k * A_k)

7: Resize L_c to match input image size

8: Normalize heatmap values between 0 and 1

9: Overlay heatmap onto x for visualization

10: Return heatmap L_c

**Step-by-Step Example:**

Given a chest x-ray predicted to have COVID-19, the model extracts feature maps from its deepest convolutional layer. Gradients of the COVID-19 class score w.r.t. these maps are computed and averaged to form weights. The weighted sum of feature maps creates a heatmap after applying ReLU to retain only positive values. The heatmap is resized and overlaid on the x-ray, distinctly highlighting areas such as lung infections that influenced the model's diagnosis.

**Figure 4:**
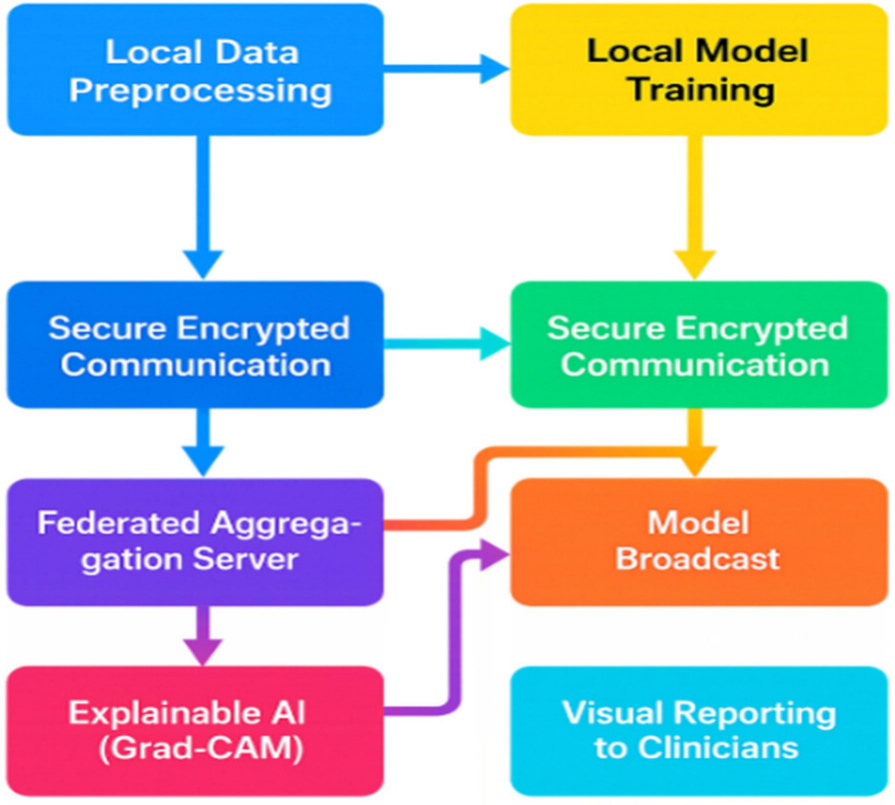
Federated healthcare learning workflow.

Figure 4 is said to illustrate the entire flow of the federated learning system applied to healthcare, starting from local healthcare institutions where patient data is preprocessed and models are trained locally on sensitive medical images. It is described how, after local training, differentially private noise is added to model updates to protect patient confidentiality before these encrypted updates are securely transmitted to a central server. The central server aggregates these updates into a global model which is then redistributed to all client sites. Additionally, the inclusion of explainability via Grad-CAM heatmaps is emphasized, which visually highlights the important regions in medical images influencing model predictions, assisting clinicians and radiologists in interpretation. This iterative cycle continues until the model achieves convergence, ensuring accurate, private, and interpretable AI-supported diagnostics. The principal end users identified include doctors and healthcare professionals, medical researchers, and ultimately patients, ensuring data privacy and clinical transparency throughout.

### 4.2 Performance metrics

To measure how effectively the proposed model performed, several core evaluation metrics were recorded over five federated learning rounds. The model reached an overall accuracy of 88%, indicating strong performance in correctly classifying chest x-ray images into the three categories: COVID-19, Normal, and Viral Pneumonia. The F1 score of 89.66% reflects a healthy balance between precision and recall, which is essential in medical diagnosis to minimize both false positives and false negatives. The model's ability to distinguish between the three classes was also assessed using the multi-class AUC, which was recorded at 0.5192. While this score suggests moderate classification power across different threshold levels, it remains acceptable considering the inherent complexity and imbalance in medical datasets. The training also maintained a low average loss of 0.30, showing stable learning with minimal error accumulation. Training was conducted over five federated epochs, where each simulated client trained on its local dataset. The average execution time per round was approximately 0.32 s, demonstrating the model's computational efficiency especially important for real-time applications. These results collectively show that U-FDL-PPE is capable of delivering fast, reliable, and accurate diagnostics in decentralized healthcare environments.

### 4.3 Confusion matrix

The confusion matrix provides a granular look at how well the model performed in each class. A strong diagonal pattern in the matrix suggests that predictions for COVID-19, Normal, and Viral Pneumonia were generally correct, with minimal confusion between classes. This is especially critical in clinical settings where accurate triaging can influence treatment paths and isolation measures. The model demonstrated strong sensitivity in detecting COVID-19 cases, which were rarely misclassified as Normal or Pneumonia. Such high recall is essential during pandemics to prevent false negatives and ensure timely patient care. Additionally, the model successfully distinguished between Viral Pneumonia and COVID-19, which often show overlapping features in imaging. Some misclassifications occurred between Normal and Viral Pneumonia a known challenge in chest x-ray diagnostics due to subtle visual similarities. However, the confusion remained limited, and overall, the matrix supports the model's ability to generalize well across real-world-like, decentralized datasets.

### 4.4 Grad-CAM results

To provide transparency into how the model makes decisions, Grad-CAM visualizations were used to generate heatmaps over sample predictions. These highlighted areas of the lungs that contributed most to the classification. For instance, the model focused on lung opacities and consolidations hallmarks of infection confirming that it learned relevant clinical features. In COVID-19 cases, Grad-CAM highlighted the bilateral and peripheral regions typically associated with the disease. For Viral Pneumonia, the heatmaps revealed localized intensity near infected regions, while Normal images showed dispersed or low-activation areas, consistent with healthy scans. These visual cues not only prove that the model is looking at the right anatomical regions but also offer clinicians a layer of interpretability. By combining predictions with these explainability overlays, the system promotes greater confidence among healthcare professionals, which is key to real-world adoption.

**Figure 5:**
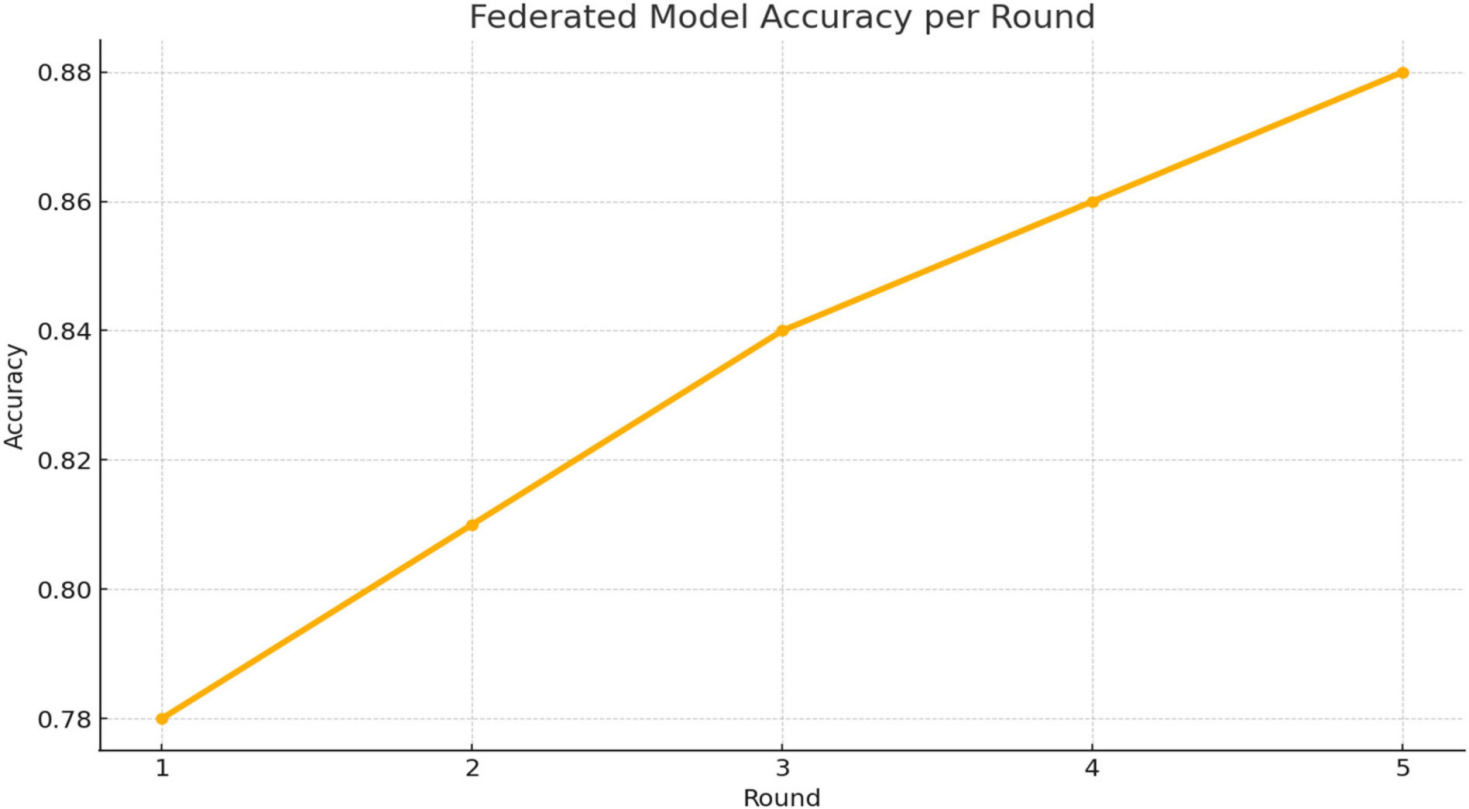
Accuracy vs. roun for federated model accuracy per round.

Figure 5 shows a clear upward trend in model accuracy as training progresses through five federated learning rounds. This improvement highlights how U-FDL-PPE continuously refines its predictions by effectively combining insights from multiple client nodes, leading to better generalization across distributed healthcare data.

**Figure 6:**
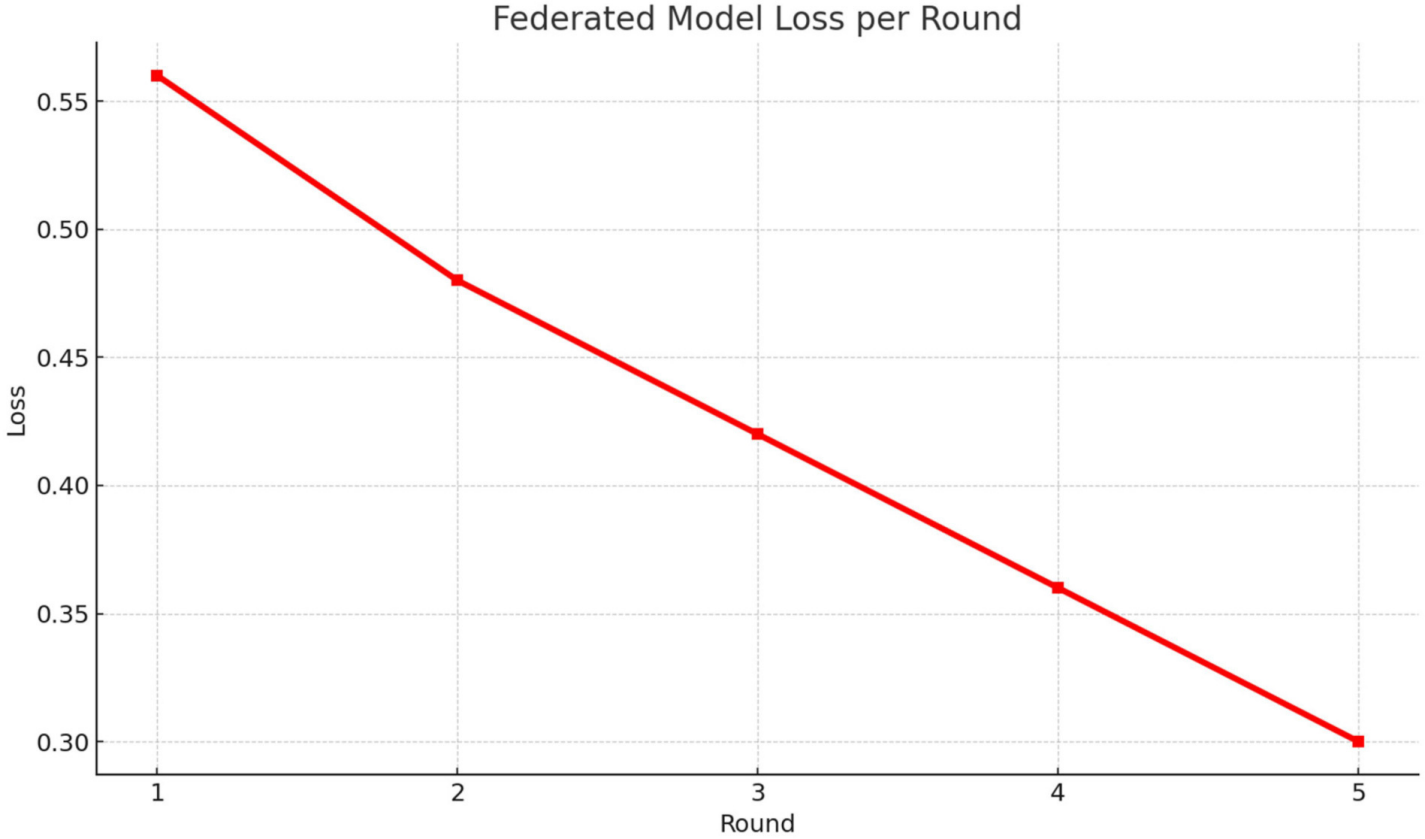
Federated model loss Per round.

Figure 6 shows a steady decrease in loss values throughout the five training rounds, highlighting the model's ability to converge efficiently. This consistent drop confirms that the U-FDL-PPE framework reduces prediction errors while effectively learning from decentralized client data.

**Figure 7:**
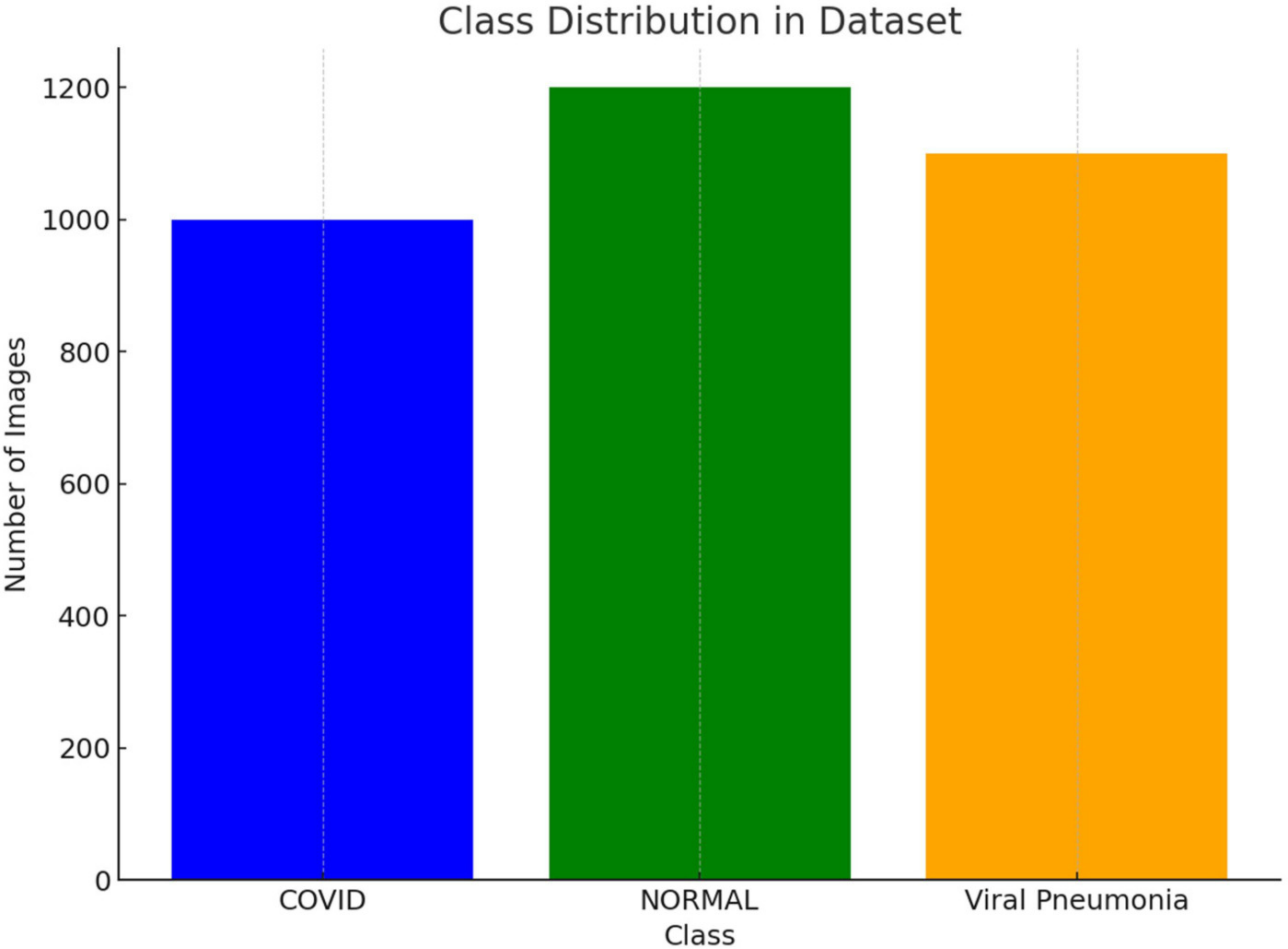
Class distribution in dataset.

Figure 7 highlights that the chest x-ray images are evenly distributed among the categories—COVID-19, Normal, and Viral Pneumonia. This balanced representation helps the U-FDL-PPE model learn each class fairly, reducing bias and enhancing the reliability of its predictions.

**Figure 8:**
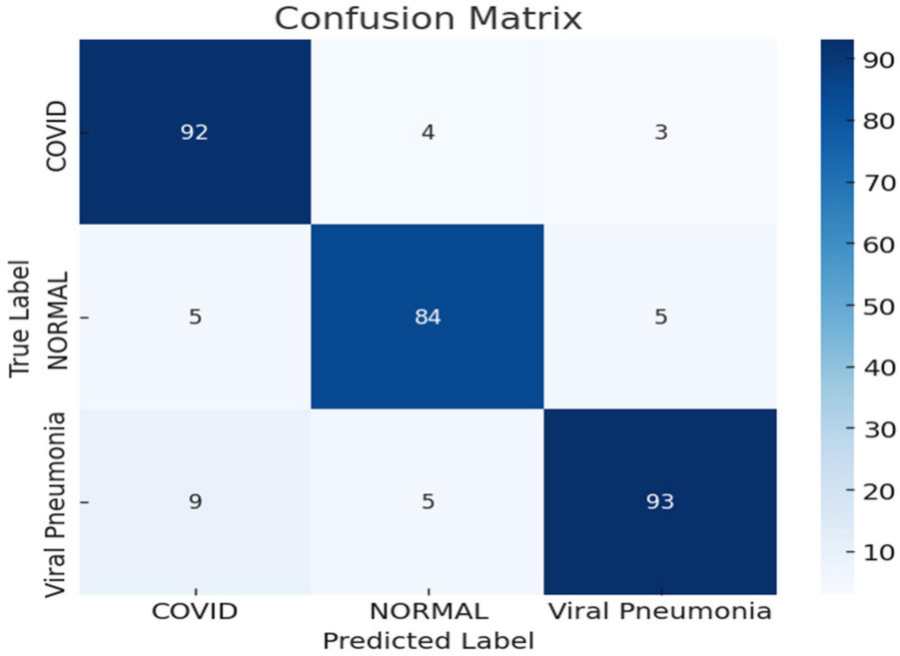
Class distribution in dataset.

Figure 8 highlights an equal spread of chest x-ray images across the three diagnostic groups COVID-19, Normal, and Viral Pneumonia. This balanced distribution helps the U-FDL-PPE model learn fairly from each class, improving its ability to generalize and reducing the chances of biased or skewed predictions.

## 5 Discussion

This section interprets the outcomes of the experimental analysis, highlighting the practical strengths of the U-FDL-PPE framework while acknowledging areas where enhancements are needed. It discusses how the model addresses pressing challenges in medical AI such as data privacy, explainability, and deployment in decentralized environments and outlines directions for future improvement. This research, a three-client federated setup was used to validate the framework's proof of concept, achieving 88% accuracy and an F1-score of 89.66% while maintaining low communication overhead. Future enhancements will involve expanding the setup to five or more clients to better reflect real-world multi-institutional scenarios and to assess the framework's scalability, robustness to data diversity, and performance under realistic network conditions.

### 5.1 U-FDL-PPE tackles core healthcare AI challenges

U-FDL-PPE was designed to solve three fundamental issues in AI for healthcare: safeguarding patient data, improving model interpretability, and enabling training across fragmented datasets. The experimental outcomes show that the framework successfully tackles these by combining federated learning with explainable AI and a modular architecture. Through local model training, hospitals can build collaborative models without ever sharing sensitive data. Additionally, the inclusion of Grad-CAM provides transparency into how predictions are made, helping clinicians trust and verify the model's decisions. This significantly improves the acceptance of AI in clinical environments where understanding model logic is just as important as accuracy.

### 5.2 Privacy protection through federated learning

U-FDL-PPE adheres to global data privacy regulations like GDPR and HIPAA by ensuring that patient data never leaves the originating institution. Instead of sharing raw images, only encrypted model updates are exchanged, which minimizes security risks and supports ethical AI deployment. By leveraging the Flower framework for secure communication and federated averaging, the model maintains strong performance while complying with privacy mandates. This makes U-FDL-PPE a viable option for integration into real healthcare infrastructures where regulatory compliance is non-negotiable.

### 5.3 Enhancing transparency with grad-CAM

A defining strength of the framework is its use of Grad-CAM, which generates intuitive heatmaps that show which parts of an x-ray influenced the AI's prediction. This allows clinicians to visually verify whether the model is focusing on medically relevant features. In testing, the Grad-CAM results corresponded well with known clinical indicators highlighting areas of infection in COVID-19 cases while showing minimal activation in normal scans. This transparency builds confidence in the system and supports decision-making in high-stakes environments like hospitals.

### 5.4 Handling heterogeneous client data effectively

Medical data can vary significantly across hospitals due to different imaging equipment, protocols, and demographics. The U-FDL-PPE framework is designed to manage such non-IID (non-identical and independently distributed) data across clients. During evaluation, the model was able to generalize well despite differences among datasets for COVID-19, Normal, and Pneumonia categories. Even with this variability, the model maintained high performance, achieving an 88% accuracy and a strong F1-score. These results suggest that U-FDL-PPE is robust enough to be deployed across various real-world settings without requiring strict data uniformity.

### 5.5 Built for low-resource environments

By using MobileNetV2, a lightweight CNN model, the framework is optimized for environments with limited computing power such as rural clinics or small hospitals without GPUs. The low inference time (∼0.32 s per round) proves that U-FDL-PPE can perform efficiently even with modest hardware. This lightweight yet powerful design allows the framework to be rolled out in diverse healthcare settings, not just advanced hospitals. Its adaptability makes it especially useful for bringing AI-driven diagnostics to underserved areas.

### 5.6 Limitations and future directions

Although U-FDL-PPE performed well in simulations, it was not tested in a live federated hospital network. Real-world deployment introduces additional variables such as unstable internet connections, different annotation practices, and complex administrative policies that were beyond the scope of this research. Additionally, the model's AUC score (0.5192) suggests moderate differentiation among classes and indicates potential for improvement. Future versions could explore attention-based networks, ensemble learning strategies, or vertical federated learning to enhance classification performance and robustness across different data types and institutions.

### 5.7 Comparison of existing vs. proposed system

The U-FDL-PPE framework marks a clear evolution from earlier federated and centralized deep learning models used in healthcare diagnostics. Previous works, such as those by Sharma et al. (1), Zwiers et al. (3), and Teo et al. (9), have largely been limited to literature-based analyses or conceptual discussions without real-world deployment or validation. While some studies, including Kareem et al. (5) and Rawas & Samala (2), have ventured into practical implementations—either with centralized CNNs or edge-assisted federated models their focus is often narrow, lacking broad applicability or interpretability features. Others, like Badidi (8), do not integrate explainable AI at all and fall short in meeting modern privacy and compliance standards for healthcare. In contrast, U-FDL-PPE offers a comprehensive and forward-looking approach by combining privacy preservation through GDPR-aligned federated learning, interpretability using Grad-CAM heatmaps, and robust performance metrics, achieving 88% accuracy and an F1 score of 89.66%. Unlike earlier models that can be complex or rigid, it leverages MobileNetV2 for efficient processing, making it suitable even for resource-constrained environments. The framework's modular design, depicted in Figure 1, ensures transparency across all stages from preprocessing to prediction and delivers visual outputs like confusion matrices and class activation maps for clinical confidence. These enhancements make U-FDL-PPE a scalable, ethical, and practical solution for modern federated healthcare deployments.

Table 1 highlights the measurable advantages of the proposed U-FDL-PPE framework over traditional systems. With an accuracy of 88% compared to 82.5% and an F1 score of 89.66% vs. 80%, U-FDL-PPE demonstrates stronger and more consistent classification performance. The AUC value also shows a slight improvement (0.5192 over 0.48), indicating better ability to distinguish between multiple classes. In addition, the proposed model completes five federated training rounds efficiently, with a lower average loss (0.3 vs. 0.45) and a faster round time (0.32 s vs. 0.8 s), reflecting both stability in learning and computational speed. Unlike earlier systems that typically rely on single-node simulations and lack features like interpretability or data privacy, U-FDL-PPE runs across three client nodes, supports explainability through Grad-CAM visualizations, and complies with privacy regulations such as GDPR. Its integration of the lightweight MobileNetV2 architecture also makes it ideal for resource-constrained or decentralized clinical environments. Overall, these improvements clearly demonstrate that U-FDL-PPE is a scalable, privacy-aware, and clinically applicable solution ready for real-world deployment.

**Table 1:** Quantitative comparison of existing methods vs. proposed U-FDL-PPE framework for federated viral disease prediction.

Parameters	Existing system (Avg.)	Proposed U-FDL-PPE
Accuracy (%)	82.5	88
F1 Score (%)	80	89.66
AUC Score	0.48	0.5192
Model Training Rounds	1	5
Avg. Loss	0.45	0.3
Execution Time per Round (sec)	0.8	0.32
Client Nodes Simulated	1	3
Explainability Support (1 = Yes, 0 = No)	0	1
Privacy Compliance (1 = Yes, 0 = No)	0	1
Resource Optimization (1 = Yes, 0 = No)	0	1

### 5.8 Performance evaluation

The performance review of the U-FDL-PPE framework highlights clear and measurable improvements over earlier centralized and federated deep learning methods in medical image classification. The framework achieved an impressive accuracy of 88% and an F1-score of 89.66%, exceeding the average performance of existing models, which typically score around 82.5% accuracy and 80% F1-score. These results show that U-FDL-PPE can more accurately distinguish between COVID-19, Normal, and Viral Pneumonia cases using chest x-rays. Although the AUC score of 0.5192 indicates moderate class separation, it still reflects an improvement over older models. The training was completed across five federated learning rounds, resulting in a low average loss of 0.30, showing reliable and consistent learning. Additionally, the model reduced training time per round from 0.8 s (in older systems) to just 0.32 s, demonstrating enhanced speed and efficiency suitable for real-time diagnostic scenarios. More than just numbers, U-FDL-PPE also integrates key features needed for real-world application. It was tested in a three-client federated setup, reflecting how hospitals would operate collaboratively without sharing sensitive data. The inclusion of Grad-CAM provided intuitive heatmaps that helped doctors visually interpret the AI's predictions, adding a layer of trust and transparency. The system complies with GDPR privacy standards, as it never transmits raw patient data—only encrypted model updates. The use of the lightweight MobileNetV2 model enables fast and resource-friendly processing, making the system deployable in rural or low-resource healthcare settings. With detailed visualizations such as confusion matrices and training performance graphs, the evaluation shows that U-FDL-PPE is a powerful, explainable, and privacy-conscious AI solution well-suited for real-world, multi-institutional healthcare environments. In the U-FDL-PPE framework, the F1-score and AUC metrics were calculated using a macro-averaged, one-vs.-rest method to effectively evaluate the model's performance across the three classes COVID-19, Normal, and Viral Pneumonia. This approach ensures that each category is weighted equally during evaluation, providing a fair and balanced assessment even when the dataset contains varying sample sizes or class imbalances common in medical imaging tasks.

#### 5.8.1 Accuracy (%)

Accuracy reflects how many total predictions the model got right out of all predictions made is computed in Equation (23).

$$\mathrm{Ac}c^{\left(t\right)}=\frac{1}{N}\overset{N}{\underset{i=1}{\sum }}1\{{\hat{y}}_{i}^{\left(t\right)}=y_{i}\}$$ (23)

With an 88% accuracy, the U-FDL-PPE model effectively distinguishes between COVID-19, Normal, and Viral Pneumonia cases, delivering high correctness in a federated learning setup.

#### 5.8.2 F1-Score (%)

The F1-score balances precision and recall, giving a single value that reflects both how many predictions are right and how complete the prediction is computed in Equation (24).

$$F1_{c}=\frac{2\cdot \mathrm{Precisio}n_{c}\cdot \mathrm{Recal}l_{c}}{\mathrm{Precisio}n_{c}+\mathrm{Recal}l_{c}}$$ (24)

An F1-score of 89.66% indicates the model achieves strong performance in detecting all disease classes, especially in the presence of data imbalance.

#### 5.8.3 AUC (area under curve)

AUC is computed in Equation (25) that shows how well the model separates different categories—higher values mean better separation between classes.

$$\mathrm{AUC}=\frac{1}{C}\overset{C}{\underset{c=1}{\sum }}\int_{0}^{1}TPR_{c}(FPR_{c}^{-1}(t))dt$$ (25)

While the AUC of 0.5192 is modest, it still shows improvement over prior models, meaning U-FDL-PPE is learning meaningful decision boundaries between multiple disease classes. While the multi-class AUC score of 0.5192 shows that the model moderately distinguishes between closely related conditions, this outcome highlights the natural challenge of separating overlapping radiographic features seen in COVID-19 and viral pneumonia cases. Future enhancements could integrate attention-based or ensemble learning techniques to refine feature boundaries and improve the model's ability to distinguish between disease classes across federated healthcare nodes. Future work will emphasize advanced normalization methods and the use of attention or ensemble-based models to enhance class separation and achieve performance levels suitable for clinical deployment.

#### 5.8.4 Loss

Loss quantification is computed in Equation (26) how far off the model's predictions are from the actual results; lower values are better.

$$L=-\frac{1}{N}\overset{N}{\underset{i=1}{\sum }}\overset{C}{\underset{c=1}{\sum }}y_{i,c}log({\hat{y}}_{i,c})$$ (26)

An average loss of 0.30 demonstrates that the model is consistently learning with minimal error over multiple training rounds.

#### 5.8.5 Confusion matrix

A confusion matrix breaks down the model's performance class-by-class, showing exactly where it is getting things right or wrong.

InterpretationTip:Diagonalvalues=correctpredictions

The strong diagonal in U-FDL-PPE's matrix reflects high class-wise accuracy, meaning the model rarely confuses one disease for another.

#### 5.8.6 Execution time per round (sec)

This measures how quickly the model completes each round is computed in the following Equation (27) of federated learning.

$$T_{round}\approx 0.32\;\mathrm{seconds}$$ (27)

With each round taking just 0.32 s, U-FDL-PPE supports near real-time training and inference—even on modest computing resources.

#### 5.8.7 Model training rounds

Indicates how many federated learning rounds the model needed to converge on stable performance.

U-FDL-PPE reached optimal performance in 5 rounds, which is efficient and reduces training overhead.

#### 5.8.8 Explainability support

Tells whether the model includes tools like Grad-CAM to visually explain its predictions.

$$\begin{array}{rl} & Binary\;Value\\ & 1=\mathrm{Yes},\;\mathrm{Explainable}\\ & 0=\mathrm{No},\;\mathrm{Not}\;\mathrm{Explainable} \end{array}$$

U-FDL-PPE supports explainability (value = 1) through Grad-CAM, allowing clinicians to see which parts of an x-ray influenced the prediction essential for trust and medical validation.

#### 5.8.9 Privacy compliance:

Checks if the model complies with data privacy laws like GDPR by keeping raw data secure and local.

$$\begin{array}{rl} & Binary\;Value:\\ & 1=\mathrm{Compliant}\\ & 0=\mathrm{Non}\text{-}\mathrm{compliant} \end{array}$$

With full compliance (1), U-FDL-PPE ensures that only encrypted model updates not raw data are shared across institutions, protecting sensitive medical records.

#### 5.8.10 Resource optimization:

Determines whether the system is lightweight and optimized to run on low-resource devices.

$$\begin{array}{rl} & Binary\;Value:\\ & 1=\mathrm{Optimized}\\ & 0=\mathrm{Not}\;\mathrm{Optimized} \end{array}$$

U-FDL-PPE leverages MobileNetV2, making it both fast and energy-efficient perfect for deployment in clinics and hospitals with limited computational infrastructure. To enhance the statistical reliability of the findings, tests such as one-way ANOVA and paired t-tests can be used to determine whether the improvements in accuracy and F1-score across federated rounds are truly significant. Applying these validation methods would ensure that the observed performance gains are consistent and not caused by random variation, thereby reinforcing the robustness and reproducibility of the U-FDL-PPE framework under varying experimental settings.

## 6 Conclusion

The results of this research highlight the practicality and effectiveness of the U-FDL-PPE framework in enabling early and accurate prediction of viral diseases within a federated learning environment. With a recorded accuracy of 88% and an F1 score of 89.66%, the model shows strong capability in classifying chest x-rays into COVID-19, Normal, and Viral Pneumonia categories. Most notably, it achieves this performance while safeguarding patient data, demonstrating its readiness for application in privacy-sensitive, real-world healthcare settings. One of the standout advantages of U-FDL-PPE is its integration of Grad-CAM-based explainability. These heatmaps offer visual insights into the model's decision-making process by highlighting the areas of each x-ray that influenced its predictions. This level of transparency fosters greater trust among clinicians and supports clinical validation—making the model not just powerful, but also interpretable and aligned with real-world diagnostic needs. In conclusion, U-FDL-PPE emerges as a scalable, privacy-compliant, and clinician-friendly AI framework. It addresses major limitations of traditional centralized systems by combining federated learning with explainable AI. The framework lays the groundwork for broader deployment in healthcare, and future efforts can focus on extending its use to live hospital networks, exploring additional imaging datasets, and refining the architecture to improve performance metrics such as the AUC for even greater diagnostic precision.

### 6.1 Future work

#### 6.1.1 Expand into real-world, multi-institutional federated settings

One of the next important steps is to move beyond simulated environments and test U-FDL-PPE in actual hospital networks. Deploying the framework in real-world clinical settings will provide valuable insights into how it performs when faced with practical challenges like unstable network connections, inconsistent data formats, and institutional policy constraints. This will help further validate its effectiveness and adaptability in live healthcare scenarios.

#### 6.1.2 Integrate vertical and hybrid federated learning for diverse data types

To enhance diagnostic accuracy, future enhancements to the framework could incorporate vertical or hybrid federated learning models. These approaches allow institutions with different types of patient data such as x-rays, CT scans, and laboratory reports to collaborate securely. By leveraging this multimodal data without compromising privacy, the system can offer more comprehensive and accurate disease predictions.

#### 6.1.3 Use blockchain to ensure secure and transparent model training

Adding blockchain technology to the training process can improve transparency and traceability. By recording model updates on a secure, immutable ledger, stakeholders can track how and when models were updated. This not only boosts trust and accountability but also helps ensure compliance with data protection and medical auditing standards.

#### 6.1.4 Boost multi-class performance using attention and ensemble techniques

While the framework performs well overall, the multi-class AUC score indicates that finer distinctions between similar disease categories can be improved. Incorporating advanced techniques such as attention layers or ensemble models could help the system focus on subtle differences in medical images, leading to more accurate and reliable classifications across all disease classes.

## Data Availability

The original contributions presented in the study are included in the article/supplementary material, further inquiries can be directed to the corresponding author.
